# Postoperative Pain Relief after Pancreatic Resection: Systematic Review and Meta-Analysis of Analgesic Modalities

**DOI:** 10.1007/s00268-021-06217-x

**Published:** 2021-06-29

**Authors:** Nasreen Akter, Bathiya Ratnayake, Daniel B. Joh, Sara-Jane Chan, Emily Bonner, Sanjay Pandanaboyana

**Affiliations:** 1 HPB and Transplant Unit, Freeman Hospital, Newcastle Upon Tyne Hospitals NHS Foundation Trust, Newcastle upon Tyne, UK; 2 Faculty of Medical Sciences, Newcastle University, Newcastle upon Tyne, UK; 3 Faculty of Medical and Health Sciences, University of Auckland, Auckland, New Zealand; 4 Perioperative and Critical Care Department, Freeman Hospital, Newcastle Upon Tyne Hospitals NHS Foundation Trust, Newcastle upon Tyne, UK; 5 Population Health Sciences Institute, Newcastle University, Newcastle Upon Tyne, UK

## Abstract

**Background:**

This systematic review explored the efficacy of different pain relief modalities used in the management of postoperative pain following pancreatoduodenectomy (PD) and distal pancreatectomy (DP) and impact on perioperative outcomes.

**Methods:**

MEDLINE (OVID), Embase, Pubmed, Web of Science and CENTRAL databases were searched using PRISMA framework. Primary outcomes included pain on postoperative day 2 and 4 and respiratory morbidity. Secondary outcomes included operation time, bile leak, delayed gastric emptying, postoperative pancreatic fistula, length of stay, and opioid use.

**Results:**

Five randomized controlled trials and seven retrospective cohort studies (1313 patients) were included in the systematic review. Studies compared epidural analgesia (EDA) (n = 845), patient controlled analgesia (PCA) (n = 425) and transabdominal wound catheters (TAWC) (n = 43). EDA versus PCA following PD was compared in eight studies (1004 patients) in the quantitative meta-analysis. Pain scores on day 2 (p = 0.19) and 4 (p = 0.18) and respiratory morbidity (p = 0.42) were comparable between EDA and PCA. Operative times, bile leak, delayed gastric emptying, pancreatic fistula, opioid use, and length of stay also were comparable between EDA and PCA. Pain scores and perioperative outcomes were comparable between EDA and PCA following DP and EDA and TAWC following PD.

**Conclusions:**

EDA, PCA and TAWC are the most frequently used analgesic modalities in pancreatic surgery. Pain relief and other perioperative outcomes are comparable between them. Further larger randomized controlled trials are warranted to explore the relative merits of each analgesic modality on postoperative outcomes with emphasis on postoperative complications.

**Supplementary Information:**

The online version contains supplementary material available at 10.1007/s00268-021-06217-x.

## Introduction

Postoperative pain after pancreatic resections is frequent [1, 2]. This may be attributed to a high incidence of preoperative pain resulting in use of analgesics prior to surgery and resection requiring extensive abdominal dissection with big incisions [2]. Inadequate pain control following any surgical procedure increases overall morbidity, hospital stay and recovery time [3, 4].

Epidural analgesia (EDA) is generally the analgesic modality of choice in pancreatic surgery and has been shown to have lower systemic complications such as pneumonia [5, 6], acute coronary syndrome, thromboembolism and renal failure[7], though at the expense of the need for vasopressors [8], excessive fluid administration [5], lengthened intensive care stay [9], and higher rates of complications such as postoperative pancreatic fistula (POPF) [8]. When EDA cannot be used, opioids via patient controlled analgesia (PCA) is often the preferred alternative. Unlike EDA, PCA does not promote hypotension, so undoubtedly is associated with a reduced need for vasopressor therapy and fluid administration [5]. More recently, transabdominal wound catheters (TAWC) have become more common in general abdominal surgery, as it has shown to be comparable to EDA in terms of pain relief with fewer complications, such as block failure and hypotension [10].

Although a variety of pain modalities have been explored for the management of postoperative pain after pancreatic surgery, the literature is generally limited to pair-wise comparisons, small study sizes and heterogeneity in their study population[2, 5, 8, 9, 11–23] making it difficult to justify routine use of one pain modality over the other. This is reflected in the recently published ERAS guidance [6] which recommends EDA for postoperative pain relief and TAWC as an alternative, however the majority of evidence for this recommendation was extrapolated from non-pancreatic surgery.

The present meta-analysis and systematic review therefore aimed to summarize and compare the efficacy of different local and regional pain relief modalities in the management of postoperative pain following pancreatic resection.

## Methods

The study protocol was registered on PROSPERO (ID: CRD42020215886).

### Literature search

MEDLINE (OVID), Embase, Pubmed, Web of Science and CENTRAL databases were searched from inception to September 2020, in accordance to the PRISMA framework [24]. The following query words were used: “pancreatectomy” OR “pancreatic resection” OR “pancreas surgery” OR “pancreas operation” OR “pancreatic enucleation” OR “pancreaticoduodenectomy” AND “analgesia” OR “anaesthesia” OR “pain control” OR “pain management” OR “postoperative pain” OR “neuroaxial” OR “narcotic” OR “opioid” OR “adjuvant” OR local/regional analgesic methods such as epidural analgesia, patient controlled analgesia, wound catheter, TAP blocks, spinal and intrathecal blocks. “Explode” and MeSH functions were used where appropriate. The search was limited to English literature.

### Inclusion and exclusion criteria

Randomised controlled trials (RCTs) and cohort studies were included if they compared two or more local or regional analgesic methods following pancreatoduodenectomy (PD) and distal pancreatectomy (DP). To qualify for inclusion in the meta-analysis, comparable studies needed to evaluate the efficacy of analgesia using a Numerical Rating Scale (NRS) or something similar, such as the Visual Analogue Score (VAS) or compare other perioperative outcomes. Studies that did not have comparable pain scores or other perioperative outcomes were included in the narrative systematic review. Where possible the pain modalities for PD and DP were evaluated separately. Studies which included minimally invasive cases (laparoscopic or robotic) or grouped different types of surgeries or pancreatic resections together were excluded, unless subgroup analysis was available.

### Data extraction

All titles and abstracts were screened independently by two authors (NA, DJ), followed by a list of articles for full text review. Relevant data was extracted and reviewed by a third author (SP). Manual screening of the reference lists in identified articles was conducted for additional papers. Authors were contacted in cases of missing data.

### Primary and secondary outcome measures

The primary outcome measures were pain scores on postoperative day 2 (POD2) and day 4 (POD4) and respiratory morbidity (pneumonia). These PODs were chosen as they were the most common days when pain scores were reported allowing a statistical comparison. Pain scores were rated on the NRS from 0–10, where 0 indicated no pain at all and 10 correlated to the worst pain possible. In articles that used VAS, these were converted to the corresponding number on the NRS [25]. The secondary outcome measures included operation time (OT), bile leak, delayed gastric emptying (DGE) [26], POPF [27], length of hospital stay (LOS), mortality and opioid use (in oral morphine equivalents (OME) or milligram morphine equivalents (MME)).

### Definitions

Pancreatectomy included open PD and DP. TAWC included transverse abdominis plane (TAP) block and quadratus lumborum (QL) block when the catheter was left in to administer post-operative pain relief and paravertebral catheter. Operating time was defined as including both anaesthetic time and duration of surgery.

### Statistical analysis

The meta-analysis was conducted in its entirety with the packages: tidyverse [28], meta [29], metaphor [30], and MetaAnalyser (Jack Bowden and Christopher Jackson, UK) 31] in R project (R Foundation for Statistical Computing, Austria 2014). A Mantel–Haenszel random effects model was utilized to perform the pairwise meta-analysis with a Hartung– Knapp adjustment. Outcomes that had 3 or more studies with an incidence of greater than 0 were included in the analysis. Where possible, outcomes from randomised and non-randomised studies were reported separately. Primary and secondary outcomes were presented using odds ratio (OR) for categorical data and standardised mean difference (SMD) for continuous data, accompanied by respective 95% confidence intervals (CI). A *p* value of <0.05 was considered significant. Heterogeneity was assessed using the *I*^*2*^ statistic; a threshold of 50% suggested moderate heterogeneity and 75% indicated substantial heterogeneity [32].

### Assessment of study quality

The quality of RCTs was evaluated using the Cochrane Risk-of-Bias tool 2.0 [33]. The Newcastle–Ottawa Scale (NOS) [34] was utilised to assess the quality of non-randomised studies.

## Results

**Fig. 1:**
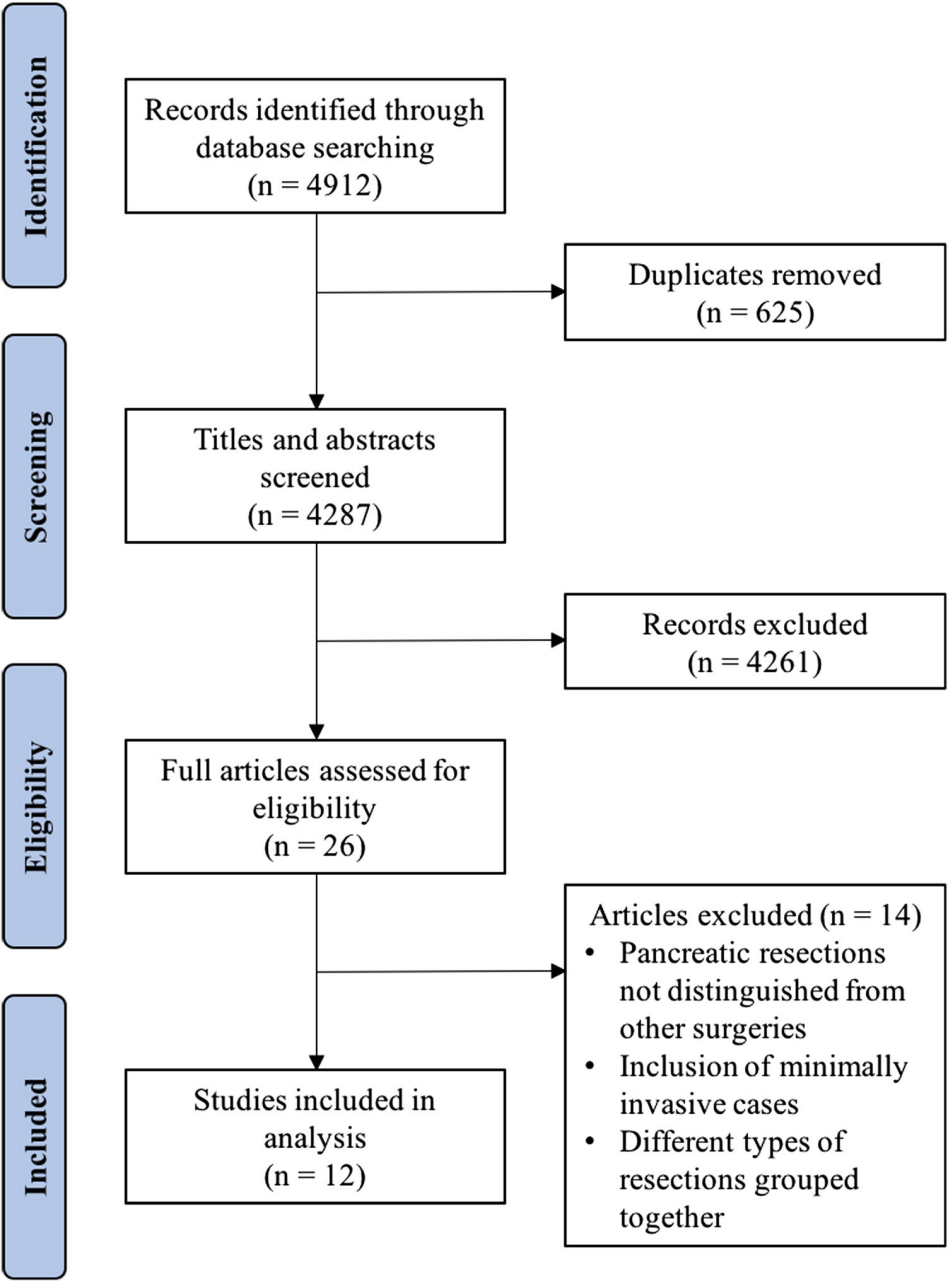
PRISMA flow diagram of screening process

The original search identified 4912 studies, of which twenty-six full text articles were screened. Following this, twelve studies [5, 8, 13, 15, 17–21, 35–37] met the inclusion criteria, of which eight studies [5, 13, 19–21, 35–37] were included in the meta-analysis. The remaining four studies [8, 15, 17, 18] were included in the narrative review (Fig. 1).

Overall, 1313 patients were included. This incorporated five RCTs [5, 8, 15, 17, 21] and seven retrospective cohort studies [13, 18–20, 35–37], published between 2020 and 2008. Studies were conducted in the USA (*n*=6), UK (*n*=1), New Zealand (*n*=1), Netherlands (*n*=1), Korea (*n*=1), Italy (*n*=1) and other parts of Europe (*n*=1) (Table 1).

**Table 1:** Study Characteristics

	Publication year	Country	Recruitment dates	Centres	Type of pancreatic Resection	Analgesic modalities compared (n)	Inclusion in meta-analysis	
RCTs								
Klotz et al. [5]	2020	Europe	2015–2017	9	PD	EDA (124)	PCA (124)	Yes
Hutchins et al. [15]	2018	USA	2012–2015	1	PD	EDA (23)	TAWC (25)	No
Mungroop et al. [8]	2016	NL	2015	Multiple	PD	EDA (18)	TAWC (18)	No
Koo et al. [17]	2016	Korea	2014–2015	1	PD	High dose PCA (53)	Low dose PCA (57)	No
Marandola et al. [21]	2008	Italy	2002–2007	1	PD	EDA (16)	PCA (24)	Yes
Cohort studies								
Kim et al. [13]	2019	USA	2014–2015	Multiple	PD	EDA (167)	PCA (43)	Yes
					DP	EDA (24)	PCA (18)	
Axelrod et al. [35]	2015	USA	2007–2011	1	PD	EDA (149)	PCA (14)	Yes
Patel et al. [18]	2014	UK	2006–2009	1	PD	Functional EDA (42)	Prematurely aborted EDA (31)	No
Shah et al. [37]	2013	USA	2007–2011	Multiple	PD	EDA (87)	PCA (15)	Yes
Choi and Schoeniger [19]	2010	USA	2004–2007	1	PD	EDA (18)	PCA^*^ (24)	Yes
Sakowska et al. [36]	2009	NZ	2005–2008	1	PD	EDA (19)	PCA (5)	Yes
Pratt et al. [20]	2008	USA	2001–2007	1	PD	EDA (127)	PCA (48)	Yes

^*^Data for no EDA group (usually received PCA); RCT, randomised controlled trial; NL, Netherlands; NZ, New Zealand; PD, pancreatoduodenectomy; DP, distal pancreatectomy; EDA, epidural analgesia; PCA, patient controlled analgesia; ITM, intrathecal morphine

Eight articles compared EDA and PCA [5, 13, 19–21, 35–37] and were included in the quantitative analysis. Other articles compared EDA and TAWC [8, 15] (*n*=2), high dose PCA and low dose PCA (*n*=1) [17] and functional EDA and prematurely aborted EDA (*n*=1) [18]. Of these, none of the included studies stated the use of an ERAS pathway in their methods section. Where applicable, two out of four studies defined POPF and one out of four studies defined DGE using the ISGPS definitions.

### EDA versus PCA

#### Pancreatoduodenectomy

Eight studies (1004 patients) compared EDA and PCA in patients undergoing PD, of which two studies were RCTs and the remaining were retrospective cohort studies.

**Fig. 2:**
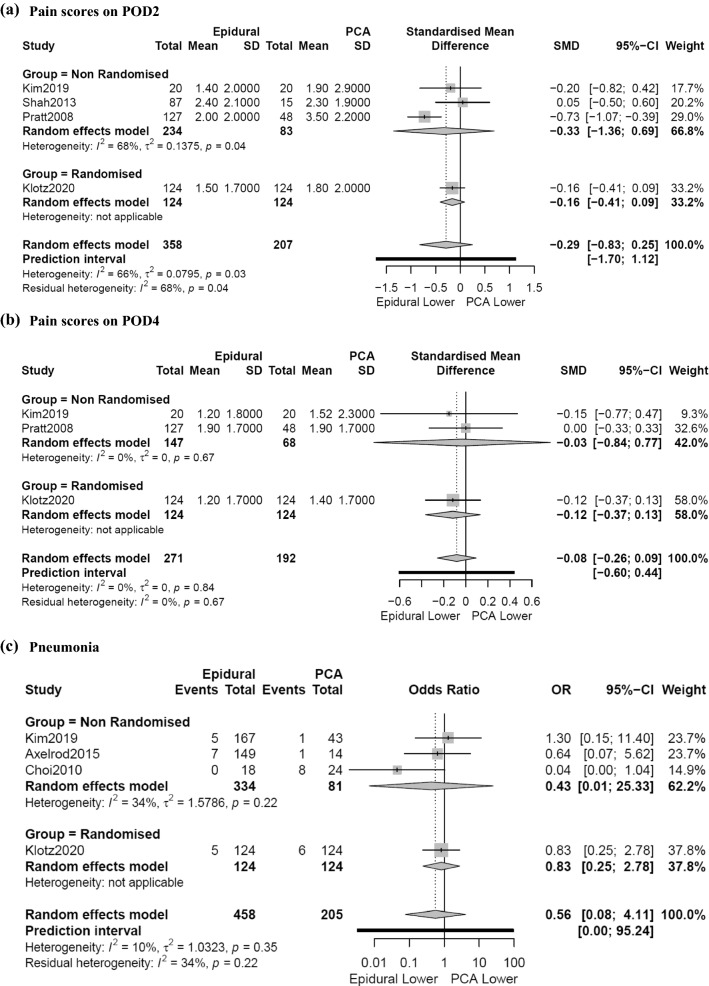
Forest plot for pain scores on POD2 (**a**) and POD4 (**b**) and pneumonia (**c**) with EA or PCA following PD

Four studies [5, 13, 20, 37] reported comparable pain scores on POD2 including a total of 565 patients (EDA: *n*=358, PCA: *n*=207) and found no significant difference in pain scores between EDA and PCA (SMD 0.29, 95% CI 0.83 to 0.24, *p*=0.19). Subgroup analysis of non-randomised studies also found no significant difference in pain scores (SMD 0.33, 95% CI 1.36 to 0.69, *p*=0.3) (Fig. 2a). Subgroup analysis of randomised studies was not possible. Three studies [5, 13, 20] reported pain scores on POD4 including a total of 463 patients (EDA: *n*=271, PCA: *n*=192). There was no significant difference in pain scores between EDA and PCA (SMD 0.08, 95% CI 0.26 to 0.06, *p*=0.18) (Fig. 2b). Subgroup analysis of non-randomised and randomised studies was not possible. On the contrary, two other studies showed lower pain scores with EDA compared to PCA on POD 1 [21] and 2 [19] respectively.

Four studies reported incidence of pneumonia in a total of 663 patients (EDA: *n*=458, PCA: *n*=205) and found no significant difference between EDA and PCA (OR 0.43, 95% CI 0.01 to 25.33, *p*=0.42). Subgroup analysis of non-randomised studies also found no significant difference (OR 0.56, 95% CI 0.08 to 4.11, *p*=0.46) (Fig. 2c). Subgroup analysis of randomised studies was not possible. Pratt et al. [20] also reported no significant difference in pneumonia between EDA and PCA (*p*=0.63).

There was no significant difference in POPF (OR 0.83, 95% CI 0.54 to 1.29, *p*=0.22) (figure supplementary (S)1a), LOS (SMD 0.09, 95% CI -0.25 to 0.42, *p*=0.38) (figure S1b), bile leak (OR 1.00, 95% CI 0.32–3.14, *p*=0.99) (figure S1c) or DGE (OR 0.89, 95% CI 0.13–6.12, *p*=0.82) (figure S1d) between EDA and PCA. Subgroup analysis of non-randomised and randomised studies was not possible. There was no significant difference in mortality (OR 0.79, 95% CI 0.29–2.16, *p*=0.55) (figure S1e) between EDA and PCA*)*. Subgroup analysis of non-randomised studies also found no significant difference in mortality (OR 0.84, 95% CI 0.14–5.19, *p*=0.85. Subgroup analysis of randomised studies was not possible.

#### Distal pancreatectomy

Kim et al. compared EDA with PCA in those that underwent DP (total: 42, EDA: 24, PCA: 18) and found no significant difference in pain scores on POD2 (*p*=0.25), POD4 (0.53), pneumonia (*p*=0.43), POPF (*p*=0.57), DGE (no incidence) or LOS (0.99).

### EDA versus TAWC

#### Pancreatoduodenectomy

Two RCTs compared EDA with TAWC [8, 15] following PD, including a total of 84 patients. The data was not suitable for a meta-analysis; hence a descriptive analysis of outcomes was undertaken. Hutchins et al. (total: *n*=48, EDA:* n*=23, paravertebral catheter: *n*=25) found no significant differences in median pain scores on POD2 (*p*=0.93) or POD4 (*p*=0.44). Mungroop et al. (total: *n*=36, EDA: *n*=18, TAWC: *n*=18) reported similar mean pain scores on POD2 (EDA: 1.2±1.1), TAWC: 0.75±1.5) P=0.30. Hutchins et al. found no significant difference in OT (*p*=0.92), LOS (*p*=0.54) or total opioid requirements in MME (*p*=0.40). Mungroop et al. found no difference in mortality (*p*=1.0).

### High dose PCA versus low dose opioid PCA

#### Pancreatoduodenectomy

Koo et al. [17], including a total of 110 patients, compared high dose remifentanil via PCA±ibuprofen (HR and HRI) and low dose remifentanil via PCA±ibuprofen (LR and LRI) following PD. There was no significant difference (*p*>0.05) in mean pain scores on POD 2 (HR: 5.2, LR: 4.9, HRI: 3.8, LRI: 4.9). No other perioperative outcomes were available.

### Functional epidural versus aborted epidural

#### Pancreatoduodenectomy

Patel et al. [18] compared functional EDA and aborted EDA following PD including a total of 73 patients. There was no data on postoperative pain scores or pneumonia, however there was no difference in LOS (functional: *n*=1.9 days, aborted *n*=2.7 days, *p*=0.48).

### Heterogeneity and risk bias

The outcomes to assess pain score on POD2 illustrated moderate heterogeneity. Five RCTs were assessed using the Cochrane Risk-of-Bias tool 2.0 (table S1). One study was assessed as having low risk, two as having some concerns and one as high risk. Seven cohort studies were assessed using the NOS scale (table S2). The average score was 7 stars. All studies scored 0 in the ‘comparability’ section which looked at comparability of cohorts based on the design or analysis. This was mainly attributed to the studies not matching their study groups.

## Discussion

The present systematic review and meta-analysis of postoperative pain management in pancreatic surgery has demonstrated that EDA provides similar level of postoperative pain relief when compared to PCA on POD2 and POD4 after both PD and DP. Furthermore, there were no significant differences in pain relief or other perioperative outcomes when comparing EDA and TAWC, high dose PCA and low dose PCA or functional EDA and aborted EDA in PD.

EDA is widely accepted as the gold standard for pain relief following major abdominal surgery [6]. However, a recent meta-analysis of RCT’s of EDA in major abdominal surgery has shown that although EDA may provide superior pain control, the perioperative outcomes are comparable to other forms of analgesia such as PCA [38]. Furthermore, patients on EDA require increased perioperative fluid administration due to sympathetic blockade [5], and have an increased incidence of perioperative complications, particularly higher POPF rate in those undergoing PD in several recent studies [8, 39–41]. In the present review, when EDA was compared with PCA, pain scores were comparable and both groups had similar postoperative complications. Although comparable data was not available on the use of postoperative fluid requirement, Klotz et al. [5] in a RCT comparing EDA with PCA showed significant weight gain and need for vasopressors with EDA, albeit with no significant increase in postoperative complications, in addition to higher failure rate with EDA (18.5%). Similarly, Simpson et al. [42] in a retrospective series, showed 31% of patients developed either hypotension or opioid toxicity after EDA in the postoperative period, albeit with improved pain scores compared to non-EDA. A more recent study using a goal-directed fluid restriction strategy with EDA during pancreaticoduodenectomy has shown lower rates of POPF and DGE [41]. The present evidence regarding the impact of volume of perioperative fluids and postoperative complications in pancreatic surgery is predominantly derived from retrospective studies and larger studies are warranted. ERAS society guidelines for pancreatic surgery suggest a high evidence level for superior pain control with EDA and a low evidence level for recommendation of EDA to reduce overall morbidity [6]. The results from the present meta-analysis suggest EDA and PCA provide similar levels of pain relief and morbidity postoperatively, however further studies are needed with predefined end-points to see the effect of EDA on POPF and morbidity following pancreatic surgery [6].

TAWC are increasingly being used in pancreatic surgery, given the perceived benefits of TAWC in major abdominal surgery [10, 43]. TAWC provides a similar level of pain relief as EDA and is associated with fewer complications [8, 10, 44]. Two studies in the present review compared EDA with TAWC with different primary and secondary outcomes, making interpretation of benefits of one analgesic modality over other difficult. No significant difference was found in OT, POPF, DGE, LOS, significant morbidity, mortality or opioid use. On the contrary, a study by Newhook et al. [12] found EDA resulted in lower opioid requirements compared to TAWC, however the pain scores in the postoperative period were similar between the analgesic modalities and failure rate was higher with EDA when compared to TAWC. Furthermore, on POD3 there was tendency trend for increased need for vasopressors after EDA with a higher proportion of patients with a postoperative rise in creatinine compared to baseline. The postoperative outcomes in all included studies were comparable between EDA and TAWC, findings similar to a recent RCT of EDA and TAWC in HPB surgery [8] which showed comparable pain relief with EDA and TAWC, however TAWC was associated with shorter anaesthetic time, lower mean cumulative vasopressor and opioid consumption. A post-hoc sensitivity analysis including only patients undergoing PD again showed non-inferiority of TAWC over EDA.

There are several limitations to the present review. The postoperative pain scores were assessed by few studies thereby meta-analysis was only possible for EDA versus PCA. The varied primary and secondary outcomes of included studies meant we could not undertake a meta-analysis of perioperative outcomes and was limited to a narrative review. Most the included studies were non-randomised, thus at risk of bias. Furthermore, there is paucity of data on patient related outcomes and a lack of data regarding the perceptions and preferences of patients. However, this is the first comprehensive review of analgesic management in patients undergoing pancreatic surgery comparing relative benefits for each analgesic modality.

For a practicing clinician, the present review summarized the available evidence on postoperative pain management after pancreatic surgery. The majority of evidence is centered around the use of EDA, PCA and TAWC, with comparable pain relief with all three analgesic modalities, in addition to a similar profile of postoperative complications. Depending on the availability of local expertise, all the above analgesic modalities provide adequate pain relief in the postoperative period. Nevertheless, there is still a lack of robust randomised evidence regarding the impact of increased fluid requirements with EDA and postoperative complications such as POPF, when compared to PCA or TAWC, as none of the trials were adequately powered to evaluate this. In addition, is it unknown which analgesic modality provides adequate pain relief when patients develop postoperative complications such as postoperative acute pancreatitis or POPF. Further high-powered RCTs are warranted to assess the relative merits of these analgesic modalities on not only postoperative pain, but postoperative outcomes with emphasis on patient related outcomes and quality of life, particularly in the setting of ERAS pathways. In addition, given the morbidity profile of PD and DP is different, future trials should aim to separate these two patient groups when evaluating postoperative outcomes.

## Supplementary Information

Below is the link to the electronic supplementary material.

Supplementary file1 (DOCX 624 kb)
