# Supplementary material for: Postoperative Pain Relief after Pancreatic Resection: Systematic Review and Meta-Analysis of Analgesic Modalities
Source: World J Surg. 2021 Jun 29;45(10):3165–73. doi: 10.1007/s00268-021-06217-x (PMC8408074; doi:10.1007/s00268-021-06217-x)
Supplement: Supplementary file 1 — Supplementary file1 (DOCX 624 kb) [file 268_2021_6217_MOESM1_ESM.docx]

Supplementary Material

Postoperative Pain Relief after Pancreatic Resection: Systematic Review and Meta-Analysis of Analgesic Modalities

Nasreen Akter^1,2^, Bathiya Ratnayake^3^, Daniel B Joh^3^, Sara-Jane Chan^1,2^, Emily Bonner ^4^, Sanjay Pandanaboyana^1,5^

^1^HPB and Transplant Unit, Freeman Hospital, Newcastle upon Tyne Hospitals NHS Foundation Trust, Newcastle upon Tyne, UK

^2^Faculty of Medical Sciences, Newcastle University, Newcastle upon Tyne, UK

^3^Faculty of Medical and Health Sciences, University of Auckland, Auckland, New Zealand.

^4^Perioperative and Critical Care Department, Freeman Hospital, Newcastle upon Tyne Hospitals NHS Foundation Trust, Newcastle upon Tyne, UK

^5^Population Health Sciences institute, Newcastle University, Newcastle Upon Tyne, UK

Correspondence:

Mr Sanjay Pandanaboyana, FRCS, MPhil

Consultant Surgeon

HPB and Transplant Unit

Freeman Hospital, Freeman Road, Newcastle upon Tyne, NE7 7DN, UK
Email: [s.pandanaboyana@nhs.net](mailto:s.pandanaboyana@nhs.net)

Journal: World Journal of Surgery

Supplementary Figures and Tables

**Fig S1** Forest plot for a) POPF, b) LOS, c) bile leak, d) DGE, e) overall mortality with EDA or PCA following PD

1. **POPF**


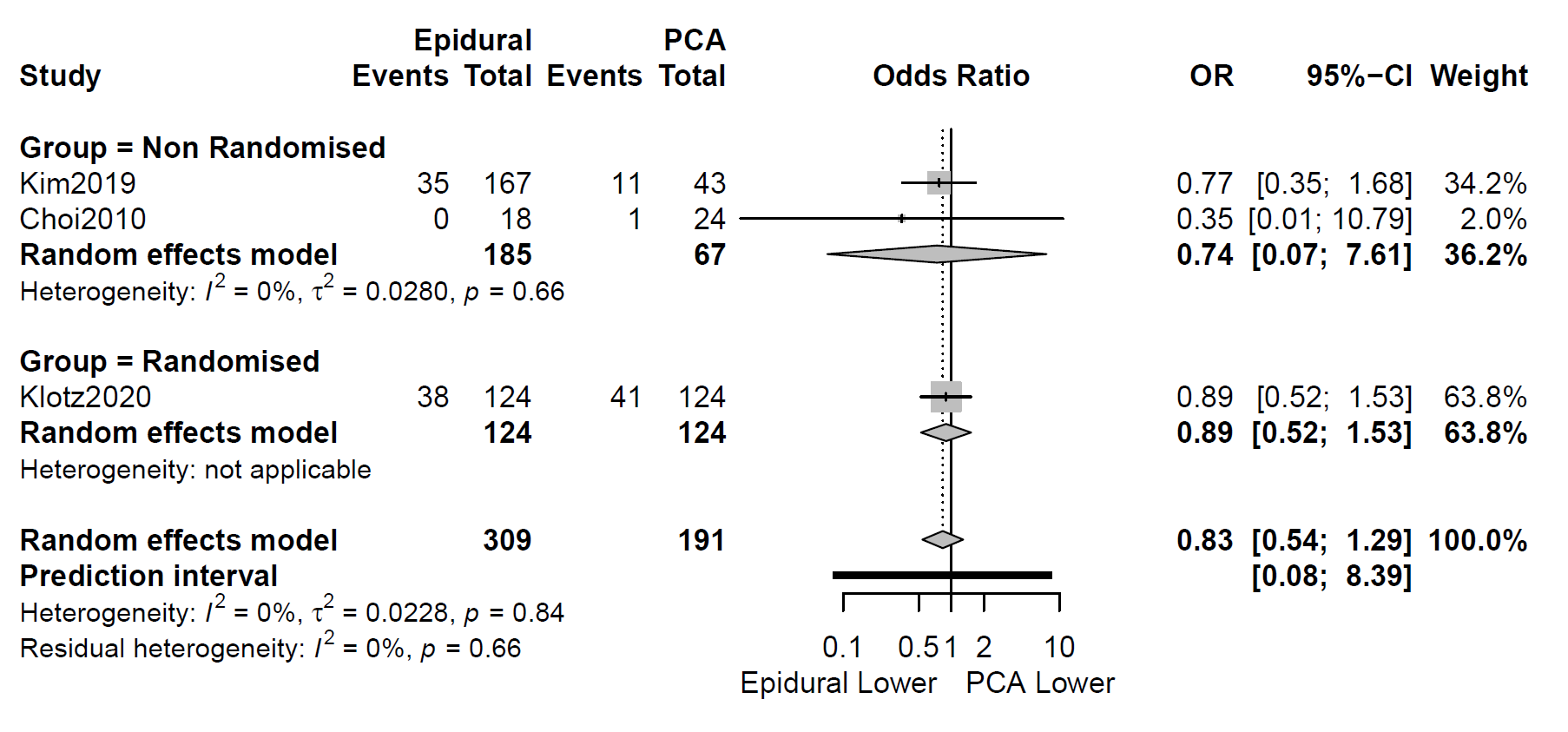


1. **LOS**


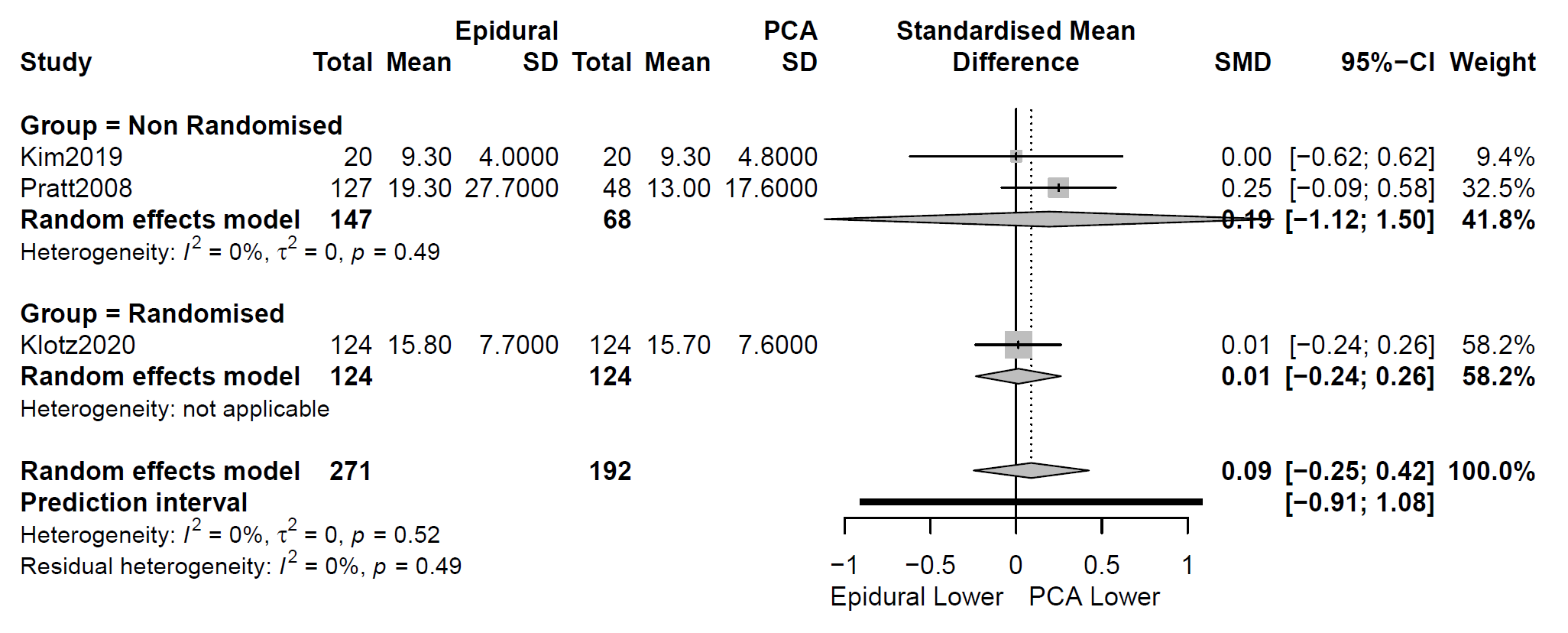


1. **Bile leak**


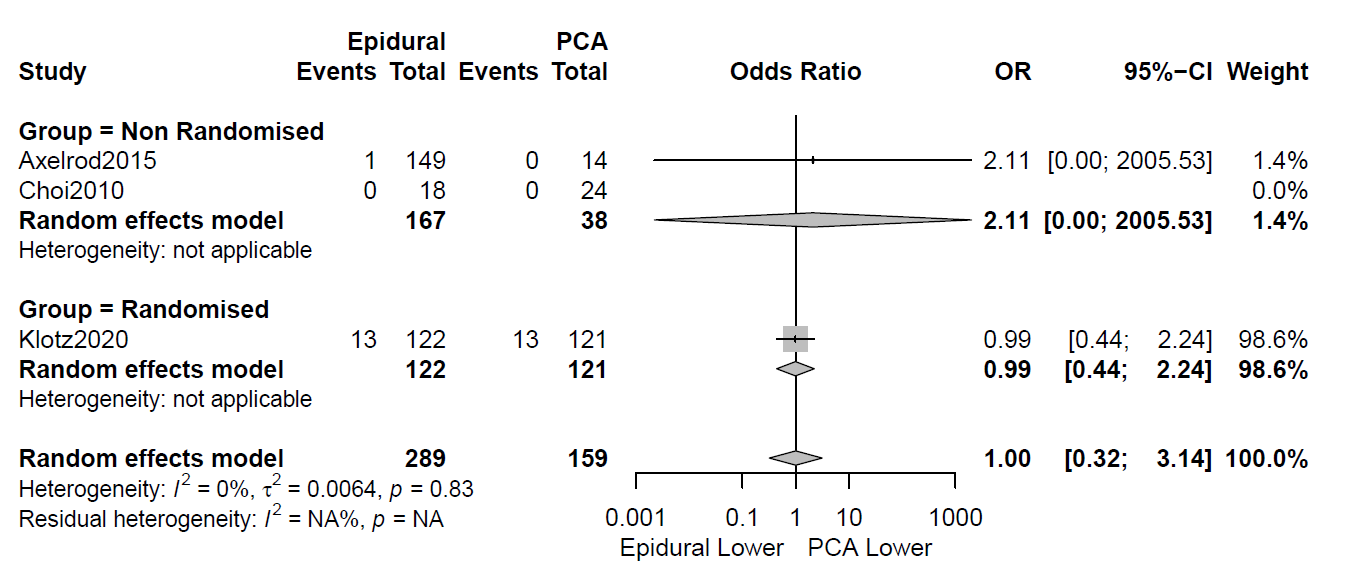


1. **Delayed gastric emptying**


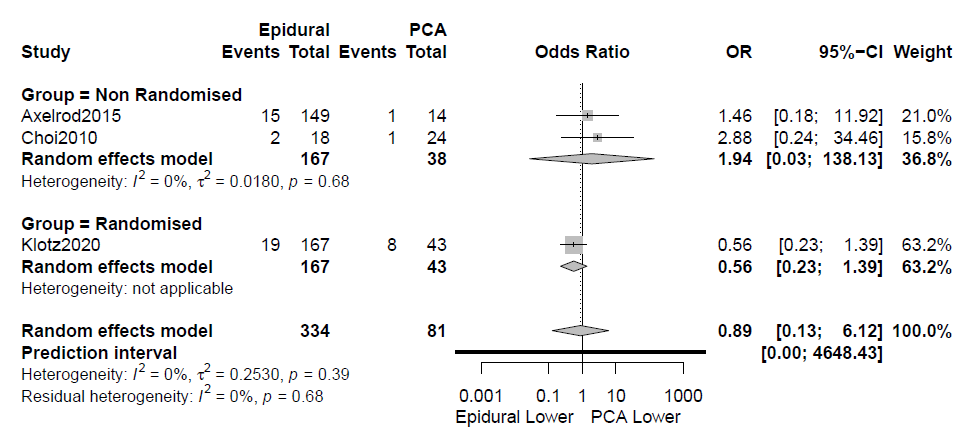


1. **Mortality**


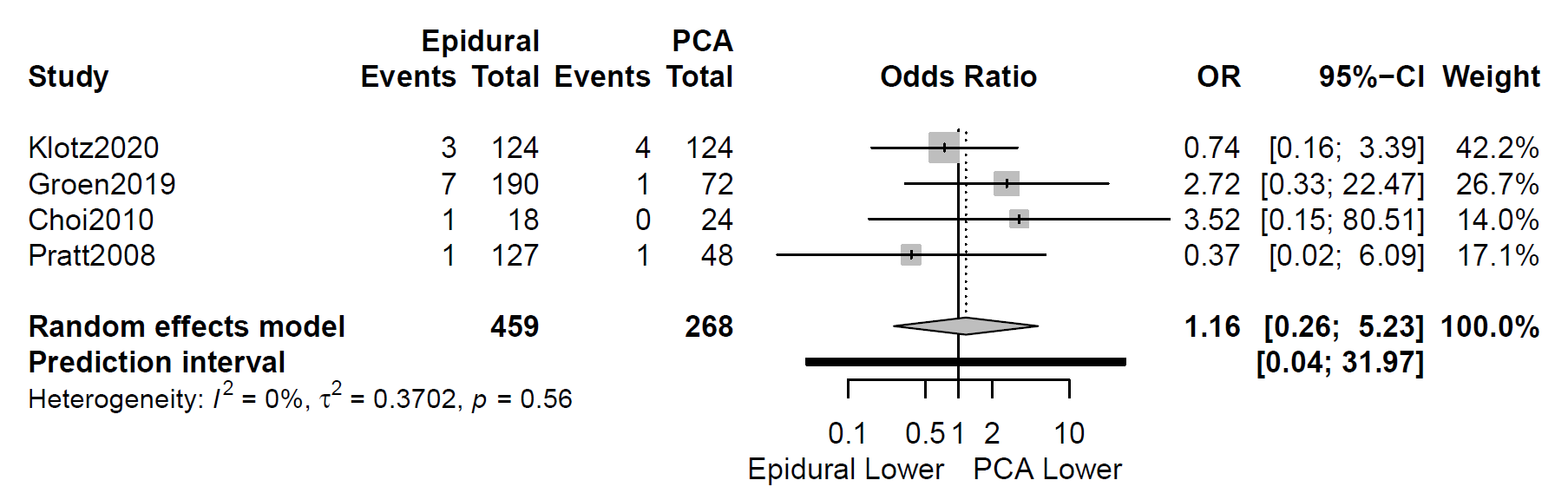


**Table S1** Summary of Cochrane Risk-of-Bias Tool 2.0 assessment of RCTs

|  | Randomisation process | Deviations from intended interventions | Missing outcome data | Measurement of the outcome | Selection of the reported result | Overall bias |
| --- | --- | --- | --- | --- | --- | --- |
| Klotz 2020 | Low risk | Low risk | Low risk | Some concerns | Low risk | Some concerns |
| Hutchins 2018 | Low risk | Low risk | Low risk | Some concerns | Some concerns | High risk |
| Mungroop 2016 | Low risk | Low risk | Low risk | Some concerns | Low risk | Some concerns |
| Koo 2O16 | Low risk | Low risk | Low risk | Low risk | Low risk | Low risk |
| Marandola 2008 | High risk | High risk | Low risk | Some concerns | Some concerns | High risk |

**Table S2** Summary of Newcastle-Ottawa Scale scores for cohort studies

| Study Name | Selection | | | | Comparability of cohorts | Outcome | | | Score (out of 9) |
| --- | --- | --- | --- | --- | --- | --- | --- | --- | --- |
|  | Exposed cohort represent-ative | Non-exposed cohort selection | Exposure ascert-ainment | Outcomes not present at start |  | Assessments | Follow up lengths | Follow up adequacy |  |
| Kim 2019 | * | * | * | * | - | * | * | * | 7 |
| Axelrod 2015 | * | * | * | * | - | * | * | * | 7 |
| Patel 2014 | * | * | * | * | - | * | * | * | 7 |
| Shah 2013 | * | * | * | * | - | * | * | * | 7 |
| Choi and Schoeniger 2010 | * | * | * | * | - | * | * | * | 7 |
| Sakowska 2009 | * | * | * | * | - | * | * | * | 7 |
| Pratt 2008 | * | * | * | * | - | * | * | * | 7 |

References

1. Kehlet H, Wilmore DW. Multimodal strategies to improve surgical outcome. Am J Surg. 2002;183(6):630-41.

2. Rockemann MG, Seeling W, Brinkmann A, et al. Analgesic and hemodynamic effects of epidural clonidine, clonidine/morphine, and morphine after pancreatic surgery--a double-blind study. Anesth Analg. 1995;80(5):869-74.

3. Chou R, Gordon DB, de Leon-Casasola OA, et al. Management of Postoperative Pain: A Clinical Practice Guideline From the American Pain Society, the American Society of Regional Anesthesia and Pain Medicine, and the American Society of Anesthesiologists' Committee on Regional Anesthesia, Executive Committee, and Administrative Council. J Pain. 2016;17(2):131-57.

4. Rawal N. Current issues in postoperative pain management. Eur J Anaesthesiol. 2016;33(3):160-71.

5. Klotz R, Larmann J, Klose C, et al. Gastrointestinal Complications After Pancreatoduodenectomy With Epidural vs Patient-Controlled Intravenous Analgesia: A Randomized Clinical Trial. JAMA Surg. 2020;155(7):e200794.

6. Lassen K, Coolsen MM, Slim K, et al. Guidelines for perioperative care for pancreaticoduodenectomy: Enhanced Recovery After Surgery (ERAS®) Society recommendations. World J Surg. 2013;37(2):240-58.

7. Amini A, Patanwala AE, Maegawa FB, et al. Effect of epidural analgesia on postoperative complications following pancreaticoduodenectomy. Am J Surg. 2012;204(6):1000-4; discussion 4-6.

8. Mungroop TH, Veelo DP, Busch OR, et al. Continuous wound infiltration versus epidural analgesia after hepato-pancreato-biliary surgery (POP-UP): a randomised controlled, open-label, non-inferiority trial. The Lancet Gastroenterology and Hepatology. 2016;1(2):105-13.

9. Boisen ML, McQuaid AJ, Esper SA, et al. Intrathecal Morphine Versus Nerve Blocks in an Enhanced Recovery Pathway for Pancreatic Surgery. J Surg Res. 2019;244:15-22.

10. Ventham NT, Hughes M, O'Neill S, et al. Systematic review and meta-analysis of continuous local anaesthetic wound infiltration versus epidural analgesia for postoperative pain following abdominal surgery. Br J Surg. 2013;100(10):1280-9.

11. Solis-Velasco MA, Ore Carranza AS, Stackhouse KA, et al. Transversus abdominis plane block reduces pain and narcotic consumption after robot-assisted distal pancreatectomy. HPB (Oxford). 2019;21(8):1039-45.

12. Newhook TE, Dewhurst WL, Vreeland TJ, et al. Inpatient Opioid Use After Pancreatectomy: Opportunities for Reducing Initial Opioid Exposure in Cancer Surgery Patients. Ann Surg Oncol. 2019;26(11):3428-35.

13. Kim SS, Niu X, Elliott IA, et al. Epidural Analgesia Improves Postoperative Pain Control but Impedes Early Discharge in Patients Undergoing Pancreatic Surgery. Pancreas. 2019;48(5):719-25.

14. Groen JV, Slotboom DEF, Vuyk J, et al. Epidural and Non-epidural Analgesia in Patients Undergoing Open Pancreatectomy: a Retrospective Cohort Study. J Gastrointest Surg. 2019;23(12):2439-48.

15. Hutchins JL, Grandelis AJ, Kaizer AM, et al. Thoracic paravertebral block versus thoracic epidural analgesia for post-operative pain control in open pancreatic surgery: A randomized controlled trial. J Clin Anesth. 2018;48:41-5.

16. Aloia TA, Kim BJ, Segraves-Chun YS, et al. A Randomized Controlled Trial of Postoperative Thoracic Epidural Analgesia Versus Intravenous Patient-controlled Analgesia after Major Hepatopancreatobiliary Surgery. Annals of Surgery. 2017;266(3):545-54.

17. Koo CH, Cho YJ, Hong DM, et al. Influence of high-dose intraoperative remifentanil with intravenous ibuprofen on postoperative morphine consumption in patients undergoing pancreaticoduodenectomy: a randomized trial. J Clin Anesth. 2016;35:47-53.

18. Patel A, Stasiowska M, Waheed U, et al. Poor analgesic efficacy of epidural analgesia in critical care patients after pancreaticoduodenectomy. Pancreas. 2014;43(3):373-9.

19. Choi DX, Schoeniger LO. For patients undergoing pancreatoduodenectomy, epidural anesthesia and analgesia improves pain but increases rates of intensive care unit admissions and alterations in analgesics. Pancreas. 2010;39(4):492-7.

20. Pratt WB, Steinbrook RA, Maithel SK, et al. Epidural analgesia for pancreatoduodenectomy: a critical appraisal. J Gastrointest Surg. 2008;12(7):1207-20.

21. Marandola M, Cilli T, Alessandri F, et al. Perioperative management in patients undergoing pancreatic surgery: the anesthesiologist's point of view. Transplant Proc. 2008;40(4):1195-9.

22. Gottschalk A, Freitag M, Steinacker E, et al. Pre-incisional epidural ropivacaine, sufentanil, clonidine, and (S)+-ketamine does not provide pre-emptive analgesia in patients undergoing major pancreatic surgery. Br J Anaesth. 2008;100(1):36-41.

23. Rockemann MG, Seeling W, Duschek S, et al. Epidural bolus clonidine/morphine versus epidural patient-controlled bupivacaine/sufentanil: quality of postoperative analgesia and cost-identification analysis. Anesth Analg. 1997;85(4):864-9.

24. Moher D, Liberati A, Tetzlaff J, et al. Preferred reporting items for systematic reviews and meta-analyses: the PRISMA statement. PLoS Med. 2009;6(7):e1000097.

25. Bijur PE, Latimer CT, Gallagher EJ. Validation of a verbally administered numerical rating scale of acute pain for use in the emergency department. Acad Emerg Med. 2003;10(4):390-2.

26. Wente MN, Bassi C, Dervenis C, et al. Delayed gastric emptying (DGE) after pancreatic surgery: a suggested definition by the International Study Group of Pancreatic Surgery (ISGPS). Surgery. 2007;142(5):761-8.

27. Bassi C, Dervenis C, Butturini G, et al. Postoperative pancreatic fistula: an international study group (ISGPF) definition. Surgery. 2005;138(1):8-13.

28. Wickham. H. Tidyverse: Easily Install and Load the ‘Tidyverse’ [Available from: <https://cran.r-project.org/web/packages/tidyverse/index.html>.

29. Guido S. Meta: An R package for meta-analysis. R News 2007;7:40–5.

30. Viechtbauer W. Conducting meta-analyses in R with the metafor package. J Stat Softw 2010;36:1–48.

31. Bowden JJ, C. . An Interactive Visualisation of Meta-Analysis as a Physical Weighing Machine 2016 [Available from: <https://cran.r-project.org/web/packages/MetaAnalyser/index.html>.

32. Higgins JP, Thompson SG, Deeks JJ, et al. Measuring inconsistency in meta-analyses. Bmj. 2003;327(7414):557-60.

33. Sterne JAC, Savović J, Page MJ, et al. RoB 2: a revised tool for assessing risk of bias in randomised trials. Bmj. 2019;366:l4898.

34. Stang A. Critical evaluation of the Newcastle-Ottawa scale for the assessment of the quality of nonrandomized studies in meta-analyses. Eur J Epidemiol. 2010;25(9):603-5.

35. Axelrod TM, Mendez BM, Abood GJ, et al. Peri-operative epidural may not be the preferred form of analgesia in select patients undergoing pancreaticoduodenectomy. J Surg Oncol. 2015;111(3):306-10.

36. Sakowska M, Docherty E, Linscott D, et al. A change in practice from epidural to intrathecal morphine analgesia for hepato-pancreato-biliary surgery. World J Surg. 2009;33(9):1802-8.

37. Shah DR, Brown E, Russo JE, et al. Negligible effect of perioperative epidural analgesia among patients undergoing elective gastric and pancreatic resections. J Gastrointest Surg. 2013;17(4):660-7.

38. Hughes MJ, Ventham NT, McNally S, et al. Analgesia after open abdominal surgery in the setting of enhanced recovery surgery: a systematic review and meta-analysis. JAMA Surg. 2014;149(12):1224-30.

39. Bruns H, Kortendieck V, Raab HR, et al. Intraoperative Fluid Excess Is a Risk Factor for Pancreatic Fistula after Partial Pancreaticoduodenectomy. HPB Surg. 2016;2016:1601340.

40. Han IW, Kim H, Heo J, et al. Excess intraoperative fluid volume administration is associated with pancreatic fistula after pancreaticoduodenectomy: A retrospective multicenter study. Medicine (Baltimore). 2017;96(22):e6893.

41. Sulzer JK, Sastry AV, Meyer LM, et al. The impact of intraoperative goal-directed fluid therapy on complications after pancreaticoduodenectomy. Ann Med Surg (Lond). 2018;36:23-8.

42. Simpson RE, Fennerty ML, Colgate CL, et al. Post-Pancreaticoduodenectomy Outcomes and Epidural Analgesia: A 5-year Single-Institution Experience. J Am Coll Surg. 2019;228(4):453-62.

43. Mungroop TH, Bond MJ, Lirk P, et al. Preperitoneal or Subcutaneous Wound Catheters as Alternative for Epidural Analgesia in Abdominal Surgery: A Systematic Review and Meta-analysis. Ann Surg. 2019;269(2):252-60.

44. Bell R, Pandanaboyana S, Prasad KR. Epidural versus local anaesthetic infiltration via wound catheters in open liver resection: a meta-analysis. ANZ J Surg. 2015;85(1-2):16-21.
